# Development and Application of CRISPR/Cas in Microbial Biotechnology

**DOI:** 10.3389/fbioe.2020.00711

**Published:** 2020-06-30

**Authors:** Wentao Ding, Yang Zhang, Shuobo Shi

**Affiliations:** ^1^Beijing Advanced Innovation Center for Soft Matter Science and Engineering, Beijing University of Chemical Technology, Beijing, China; ^2^Key Laboratory of Food Nutrition and Safety, Ministry of Education, College of Food Engineering and Biotechnology, Tianjin University of Science and Technology, Tianjin, China

**Keywords:** CRISPR/Cas, guide RNA, genome editing, gene regulation, microbial biotechnology

## Abstract

The clustered regularly interspaced short palindromic repeats (CRISPR)-associated (Cas) system has been rapidly developed as versatile genomic engineering tools with high efficiency, accuracy and flexibility, and has revolutionized traditional methods for applications in microbial biotechnology. Here, key points of building reliable CRISPR/Cas system for genome engineering are discussed, including the Cas protein, the guide RNA and the donor DNA. Following an overview of various CRISPR/Cas tools for genome engineering, including gene activation, gene interference, orthogonal CRISPR systems and precise single base editing, we highlighted the application of CRISPR/Cas toolbox for multiplexed engineering and high throughput screening. We then summarize recent applications of CRISPR/Cas systems in metabolic engineering toward production of chemicals and natural compounds, and end with perspectives of future advancements.

## Introduction

Microbial cell factories producing fuels, chemicals, and pharmaceutics are perspective production mode to replace petrol relied methods because microbial methods are usually clean and renewable. One restriction to the development of microbial producer is the slow, inefficient and arduous genomic engineering processes. The emerging toolbox based on clustered regularly interspaced short palindromic repeats (CRISPR) system have largely improved genome editing efficiency, simplified steps of multi-loci editing, and enabled fast disturbance of metabolic network. The CRISPR system is prokaryotic adaptive immune system against intruded heterologous DNA/RNA from virus or other organisms (Grissa et al., 2007a; Sorek et al., 2013). So far, the CRISPR/Cas system has been intensively adopted as toolbox for both fundamental studies and biotechnological applications for genome editing, molecular diagnosis, metabolic engineering, gene function mining, etc., in microorganisms, plants and mammals (Sander and Joung, 2014; Zhang et al., 2014; Wang H. et al., 2016; Tang and Fu, 2018; Tarasava et al., 2018; Armario Najera et al., 2019; Moon et al., 2019; Xu and Oi, 2019). In the field of microbial biotechnology, the CRISPR/Cas system has been applied for numerous model and non-model microorganisms, e.g., *Escherichia coli* (Jiang et al., 2013), *Saccharomyces cerevisiae* (DiCarlo et al., 2013), *Bacillus* (Westbrook et al., 2016), *Clostridium* (Li et al., 2016; Joseph et al., 2018), *Corynebacterium* (Jiang et al., 2017), *Lactobacillus* (Oh and van Pijkeren, 2014), *Mycobacterium* (Choudhary et al., 2015), *Pseudomonas* (Tan S. Z. et al., 2018), *Streptomyces* (Cobb et al., 2015). However, there still remains interested microorganisms that CRISPR system has not been applied, and some weakness of existing CRISPR/Cas systems needs to be overcome. This review focuses on the establishment and development of CRISPR toolbox for genome editing and gene regulation, and applications of these techniques in metabolic engineering and synthetic biology in microorganisms.

## The CRISPR/Cas System for Genome Editing

The CRISPR systems are adaptive evolved for counteracting foreign DNA or RNAs, and the systems are present in nearly half of bacteria and almost all archaea (Grissa et al., 2007b; Zetsche et al., 2015a), but absent from eukaryotes or viruses (Jansen et al., 2002). The CRISPR/Cas systems have been categorized into two classes and six major types based on the constitution of effector protein and signature genes, protein sequence conservation, and organization of the respective genomic loci (Koonin et al., 2017; Tang and Fu, 2018). Among these CRISPR systems, the Cas9 (Type II), Cas12a (previously known as Cpf1, type V) and their mutant variants are most investigated effectors, and have shown broad applicational potentials in genome editing, gene regulation, DNA detection, DNA imaging, etc. (Tang and Fu, 2018; Miao et al., 2019).

**FIGURE 1:**
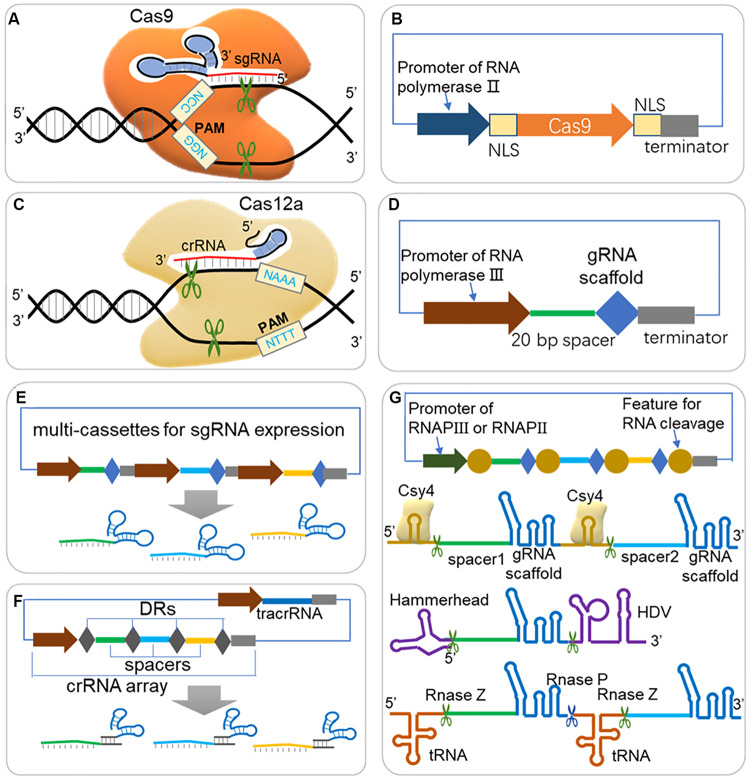
Guidelines for expression of Cas protein and sgRNA in CRISPR/Cas system. **(A)** Scheme of CRISPR/Cas9 system. The Cas9-sgRNA (or Cas9-crRNA-tracrRNA) complex binds to DNA target arising from Watson-Crick base pairing of spacer sequence, and triggers double strand break (DSB) when next to a short protospacer adjacent motif (PAM, ‘NGG’ for Cas9 from *S. pyogenes*). **(B)** Expression cassette for Cas9. For efficient targeting to nucleus in eukaryotes, the Cas9 should be fused to NLS (nuclear localization sequence) at one end or both ends. **(C)** Scheme of CRISPR/Cas12a (Cpf1) system. Cas12a triggers DSB through a similar scheme of Cas9, but depends on different PAM (‘NTTT’) and less folded crRNA, and creates a sticky end at 18–23 bases away from the PAM. **(D)** Expression cassette for sgRNA. A promoter of RNA polymerase III (RNAP III) is usually required for directing sgRNA in nucleus and with less modification. A 20 bp spacer should be well designed according to target DNA sequence for efficient editing rates and avoiding off-target effects. **(E)** Multi-sgRNA expression through multi-cassettes. Repeated elements, such as promoters, gRNA scaffold and terminators are repeated for different spacer sequences. **(F)** Multi-sgRNA expression through crRNA array and tracrRNA (HI-CRISPR system). Different spacers are separated with direct repeats (DRs) and expressed by one promoter of RNAP III. The pre-crRNA is transcribed and processed into mature crRNA by RNase III and unknown nuclease(s). The tracrRNA and Cas9 protein are complexed with mature crRNA to form the dual-RNA-guided nuclease. **(G)** gRNA multiplexing strategies. Both RNAP II and RNAP III promoter can be used for expression the sgRNA array, where sgRNAs are separated by features for RNA cleavage. RNA endonuclease Csy4 recognizes a 28 nucleotide sequence flanking the sgRNA sequence and cleaves after the 20th nucleotide. The hammerhead ribozyme and HDV ribozyme flanked the 5′ and 3′ of the sgRNA, respectively, allowing for self-cleaving production of sgRNAs, which are not dependent on the presence of an exogenous protein. Polycistronic tRNA-gRNA architecture allows the production of multiple sgRNAs by endogenous RNase P and RNase Z.

The CRISPR/Cas system can introduce a double-strand DNA break (DSB) at the specific DNA target (also called protospacer) binding by a guide RNA (gRNA) and harboring a short protospacer adjacent motif (PAM) flanked at the 3′ end of protospacer (Figures 1A,B; Garneau et al., 2010; Gasiunas et al., 2012; Jinek et al., 2012; Wang H. et al., 2016). A DSB triggers DNA repair through intrinsic cellular mechanisms, mainly including non-homologous end joining (NHEJ), which direct ligates two breaking ends with small insertions or deletions (indels); and homology-directed repair (HDR), which repair DSB according to a homologous template (Hsu et al., 2014; Doetschman and Georgieva, 2017). Considering the guide RNAs are easy to design and expressed, Cas protein can be programmed to introduce DSBs at one or more DNA targets, making CRISPR/Cas an convenient and precise platform for genome editing (Doetschman and Georgieva, 2017). Compared with similar genome editing tools such as zinc-finger nucleases (ZFNs) (Kim et al., 1996; Urnov et al., 2010) and TAL effector nucleases (TALENs) (Boch et al., 2009; Christian et al., 2010), CRISPR/Cas shows a significant advantage that it is easier to target a specific region by adjusting a 20 nt spacer sequence of gRNA, rather than producing target-specific proteins (Doetschman and Georgieva, 2017).

### Selection and Expression of Cas Protein

The CRISPR/Cas systems have been reported to have two classes and six major types, and among these types, the class 2 type II CRISPR system (CRISPR/Cas9) is currently most studied and developed as toolbox for gene editing and other applications. As shown in Figure 1A, the effector (Cas9) is activated when forming a complex with single guide RNA [sgRNA, a fusion RNA of CRISPR targeting RNA (crRNA) and trans-activating CRISPR RNA (tracrRNA) (Jinek et al., 2012)], and triggers DSB at DNA target near PAM (Mougiakos et al., 2016). The spacer part is responsible for DNA target (also called protospacer) binding, and guides the Cas9 complex for sequence specific DNA cleavage. PAM flanks the 3′ end of the protospacer, and is required for Cas9-mediated cleavage (Deveau et al., 2008; Mojica et al., 2009). The PAM of the most commonly used SpCas9 (Cas9 from *Streptococcus pyogenes*) is ‘NGG,’ which occurs once every 8 bp on average within the genome, allowing targeting on most genes of interest (Doudna and Charpentier, 2014; Hsu et al., 2014). Cas9s from different resources recognize different PAM sequences, which further expands the application of CRISPR for various genomic sequence [e.g., Cas9 from *Staphylococcus aureus* (Kleinstiver et al., 2015; Ran et al., 2015), *Streptococcus thermophiles* (Esvelt et al., 2013; Kleinstiver et al., 2015), *Neisseria meningitides* (Esvelt et al., 2013; Hou et al., 2013)]. Cas9 ‘nickase’ variant (nCas9), with mutations deactivating one nickase activity and converting the endonuclease activity of wildtype Cas9 to nickase activity, introduces a single stranded break (SSB) rather than DSB (Jinek et al., 2012; Cong et al., 2013). Generally, SSBs are repaired by HDR, not by NHEJ, thus nCas9 can be applied for precise genome editing (Standage-Beier et al., 2015). Another Cas9 mutant, the nuclease-deactivated Cas9 (dCas9), has been fused with a variety of effectors, including transcriptional activators, repressors, and epigenetic modifiers to enable sequence specific genomic regulation (Gilbert et al., 2013, 2014; Qi et al., 2013).

In 2013, the application of CRISPR/Cas9 system for genome editing was originally reported in human cells (Cong et al., 2013; Jinek et al., 2013; Mali et al., 2013b), mouse cells (Cong et al., 2013), Zebrafish (Hwang et al., 2013), *Saccharomyces cerevisiae* (DiCarlo et al., 2013), *Streptococcus pneumoniae*, and *Escherichia coli* (Jiang et al., 2013). In following studies, the CRISPR/Cas9 system has been widely applied for genome editing in numerous microorganisms, plants and animals.

As an eukaryotic model microorganism, *S. cerevisiae* was one of the earliest hosts for CRISPR/Cas9 mediated genome editing (DiCarlo et al., 2013). In order to improve genome editing efficiency, the Cas9 protein is usually highly expressed by a strong constitutive promoter [e.g., *TEF1* promoter (DiCarlo et al., 2013; Gilbert et al., 2013; Bao et al., 2015), *TDH3* promoter (Gilbert et al., 2013; Laughery et al., 2015; Jensen et al., 2017)] in a episomal CEN low copy plasmid (DiCarlo et al., 2013; Gilbert et al., 2013) or episomal 2 μ high copy plasmid (Ryan and Cate, 2014; Bao et al., 2015; Shi et al., 2016; Jensen et al., 2017). However, in some researches, expression of Cas9 with strong promoter (e.g., promoter of *TEF1*, *HXT7*, and *TDH3*) showed toxic effect to cell growth (Ryan and Cate, 2014; Generoso et al., 2016). Nevertheless, medium strength or weak promoters showed similar editing efficiency, and no significant negative impact on the strain’s growth rate. For efficient CRISPR editing rate, codon usage in heterologous organisms should be also considered to guarantee sufficient Cas9 abundance *in vivo*. In eukaryotes, Cas9 protein should be transported to nuclei to facilitate genome editing, and thus the nuclear localization sequence (NLS) should be fused to the Cas9 protein (Figure 1B). In *S. cerevisiae*, the SV40 NLS (‘PKKKRKV’) is typically fused to the N- or C-terminus of the Cas9, and two NLSs fused to one terminus or both were also applicable.

The model bacteria *E. coli* has also been intensively researched as a host for CRISPR/Cas9 mediated genome editing. However, *E. coli* lacks the NHEJ mechanism for DSB repair (Chayot et al., 2010), and is highly reliant on a native homology-directed repair system with low efficiency, challenging the DSB producing CRISPR/Cas9 system (Jiang et al., 2015). Thus, co-expression of heterologous phage-derived recombinase to improve the frequency of homologous recombination showed significant improved survival rates when CRISPR/Cas9 and gRNA expressed (Jiang et al., 2015; Pyne et al., 2015; Bassalo et al., 2016). In *E. coli*, inducible promoters were mostly used for both Cas9 and gRNA expression.

The CRISPR/Cas9 system has also been constructed with similar strategy for non-model microorganisms (Yan and Fong, 2017; Cho et al., 2018; Raschmanova et al., 2018; Wang and Coleman, 2019). Generally, species-specific strong promoters should be used for Cas9 expression, either constitutively or inducible expressed. Codon optimization should also be conducted when the Cas9 protein cannot be efficiently expressed. In eukaryotic microorganisms, NLS should be fused to Cas9 at one or both termini for cell nucleus localization. The NLS of SV40 from *S. cerevisiae* has been proven effective and applied in other yeast species. Native DNA repair types and efficiency also largely determined genome editing rate, because DSB induced by CRISPR/Cas9 can be repaired by NHEJ, resulting in indels and gene inactivation, or be repaired by HDR, resulting in precise genome editing by supplying proper DNA donors. Thus, in some organisms with both NHEJ and HDR pathways, deletion of *KU70*/*KU80* often repressed NHEJ and increased CRISPR mediate genome editing rate through HDR (Gao S. et al., 2016; Schwartz et al., 2016; Cao et al., 2018; Bae et al., 2020). However, in some organisms lacking HDR, phage-derived recombinases (RecET and λ-Red) should be co-expressed with Cas9, similar to the approaches adopted *in E. coli* (Jiang et al., 2015; Wang B. et al., 2018).

In addition to widely applied Cas9, Cas12a (also known as Cpf1) is a newly emerging Cas protein that is currently under evaluation for gene editing potential (Zetsche et al., 2015a). Cas12a is a crRNA-guided endonuclease, lacking tracrRNA compared with Cas9, and cleaves DNA at 18 nucleotides away from the PAM, resulting in a DSB with 4- to 5-nucleotide overhangs (Figure 1C; Zetsche et al., 2015a). Besides, Shmakov et al. (2015) further classified three class 2 CRISPR systems, including C2c1, C2c3, and C2c2, which further expands CRISPR toolbox for genome editing.

### Design and Expression of Guide RNA

The efficient expression of guide RNA is also critical to a CRISPR system because the spacer sequence of guide RNA is responsible for DNA target binding and thus decides the editing loci, and is closely related to on-target and off-target efficiency. Generally, one or more single guide RNAs (sgRNAs) are expressed in a CRISPR/Cas system (Figures 1D–G); but in some other cases, a crRNA matrix and a tracrRNA, instead of sgRNAs, are expressed separately for efficient CRISPR editing (Bao et al., 2015). The spacer sequence should be carefully designed, which binds to a DNA target close to a PAM sequence, and to promote editing efficiency and reduce off-target rate. A serial of studies have suggested that mismatches at the 5′ end of spacer sequence are generally better tolerated than those at the 3′ end, and especially the 8–12 bps at the 3′ end of the spacer sequence are crucial for target recognition (Cong et al., 2013; Fu et al., 2013; Hsu et al., 2013; Jiang et al., 2013; Sander and Joung, 2014). It is crucial to design gRNAs for CRISPR system, and a well-selected gRNA would minimize the risk of CRISPR-mediated DSBs at unwanted sites in genome (off-target effects) and maximize the editing efficiency at the selected site (on-target activity) (Stovicek et al., 2017). Several rules and algorithms have been proposed, and web-tools for gRNA design can help to choose best gRNAs in various species (shown in Table 1). The rules for gRNA scoring includes possible binding sites with mismatches in the spacer sequence or in the seed sequence, the GC content and poly T presence and self-complementarity (Heigwer et al., 2014; Liu et al., 2015; Naito et al., 2015; Labun et al., 2019). Except for gene editing, CRISPR-ERA and CHOPCHOP also help to design gRNAs for gene activation and repression (Liu et al., 2015; Labun et al., 2019).

**TABLE 1:** List of selected Web-sites for gRNA design in multi-species.

**Name**	**Link**	**PAM**	**Organism**	**Function**	**References**
CHOPCHOP v3	http://chopchop.cbu.uib.no	Most reported PAMs or a self-defined sequence	Over 200 genomes	Knock out/knock in/activation/repression/Nanopore enrichment	Labun et al., 2019
E-CRISPR	http://www.e-crisp.org/	Most reported PAMs	Over 50 genomes	Single design/paired designs	Heigwer et al., 2014
ATUM	https://www.atum.bio/eCommerce/cas9/input	NGG/NAG	*Homo sapiens*/*Mus musculus*/*Saccharomyces cerevisiae*/*Escherichia coli*/*Arabidopsis thaliana*		
CRISPRdirect	https://crispr.dbcls.jp/	Self-defined PAM	Over 200 species		Naito et al., 2015
CRISPR-ERA	http://crisprera.stanford.edu/	NGG	Human/mouse/rat/zebrafish/ *D. melanogaster*/ *C. elegans*/*S. cerevisiae*/ *E. coli*/*B. subtilis*	Gene editing/activation/repression	Liu et al., 2015
CC TOP	https://crispr.cos.uni-heidelberg.de	Most reported PAMs	102 species	gRNA and off-target prediction	Stemmer et al., 2015

Generally, a strong expression of gRNA is recommended for an efficient target binding and CRISPR complex activation. To express RNA without modifications added by the RNA polymerase II (RNAPII) transcription system, RNA polymerase III (RNAPIII) regulatory elements have been used for transcription of functional gRNA (Figure 1D), e.g., the *SNR52* promoter has been used in yeast (Raschmanova et al., 2018) and *U6* promoter has been used in human cells (Zhang et al., 2014; Wang H. et al., 2016). However, it is noted that some promoters require special rules of gRNA sequence, e.g., the U6 promoter or the T7 promoter require a ‘G’ or ‘GG,’ respectively, at the 5′ end of the RNA to be transcribed (Sander and Joung, 2014; Wang H. et al., 2016). Despite RNAPIII promoters are suitable for gRNA transcription, in some organisms, however, these promoters are poorly characterized. On the other hand, RNAPII promoters can also be used to express gRNAs when proper strategies are adopted (Nowak et al., 2016). A RNAPII promoter of *rrk1* and its leader RNA was used to express sgRNA by flanking a Hammerhead ribozyme on the 3′ end of gRNA (Figure 1G) in fission yeast (Jacobs et al., 2014). Another research also used RNAPII promoter but flanked the sgRNA with a 28 nucleotide hairpin at each end that is recognized by the endoribonuclease Csy4 (Figure 1G; Nissim et al., 2014). Fusion gRNAs with a hammerhead (HH) ribozyme on their 5′ end and a hepatitis delta virus (HDV) ribozyme on their 3′ end was also reported functional for RNAPII promoter (Figure 1G; Nissim et al., 2014; Weninger et al., 2016). Interestingly, fusion of sgRNA with special RNA scaffold (e.g., HDV, RNA triplex) would increase *in vivo* RNA stability and thus promote engineering efficiency (Nissim et al., 2014; Ryan and Cate, 2014).

When CRISPR/Cas system is constructed for multi-loci editing (Figures 1E–G), several strategies have been proposed to enable an efficient expression of multiple gRNAs. Multi-sgRNA expression could be achieved through multi-expression cassettes using individual promoters to control each gRNA (Figure 1E). This method was successfully demonstrated to enable multiple editing (Jakociunas et al., 2015). For another strategy, the crRNA matrix and tracrRNA were expressed separately by RNAPIII promoters, and processed into mature crRNA by RNase III and unknown nuclease(s) (Figure 1F), which also showed high gene disruption efficiency in *S. cerevisiae* (Bao et al., 2015). The tRNA-processing system, which precisely cleaves both ends of the tRNA precursor by RNase P and RNase Z (or RNase E in bacterium, Figure 1G), exists in virtually all organisms and can be broadly used to boost the targeting capability and editing efficiency of CRISPR/Cas systems (Xie et al., 2015; Port and Bullock, 2016; Qi et al., 2016; Ding et al., 2018; Zhang et al., 2019). Single or multiple gRNAs can be expressed by one promoter but separated by tRNA scaffolds [e.g., a 71 bp long pre-tRNA^Gly^ (Xie et al., 2015; Zhang et al., 2019)].

It is costly and time consuming for the sub-cloning of plasmids used for multi-loci editing, and some strategies could be taken for saving cloning time or improving editing efficiency. Gibson assembly, Golden gate cloning and USER cloning have showed high rates in multi DNA fragments assembly, which simplifies cloning steps for multiple gRNA expression cassettes, and thus saves the processing time (Bao et al., 2015; Shi et al., 2016; Smith et al., 2016; Jensen et al., 2017; Zhang et al., 2019). Meanwhile, *in vivo* homologous recombination has been reported for rapid assembling a certain plasmid backbone and PCR cassettes bearing sgRNAs in some yeast species (*S. cerevisiae* and *K. lactis*), thus saving cloning steps for high-efficiency engineering (Horwitz et al., 2015; Generoso et al., 2016; Reider Apel et al., 2017).

### DNA Repair and Donor Design for DNA Deletion, Insert and Mutation

**FIGURE 2:**
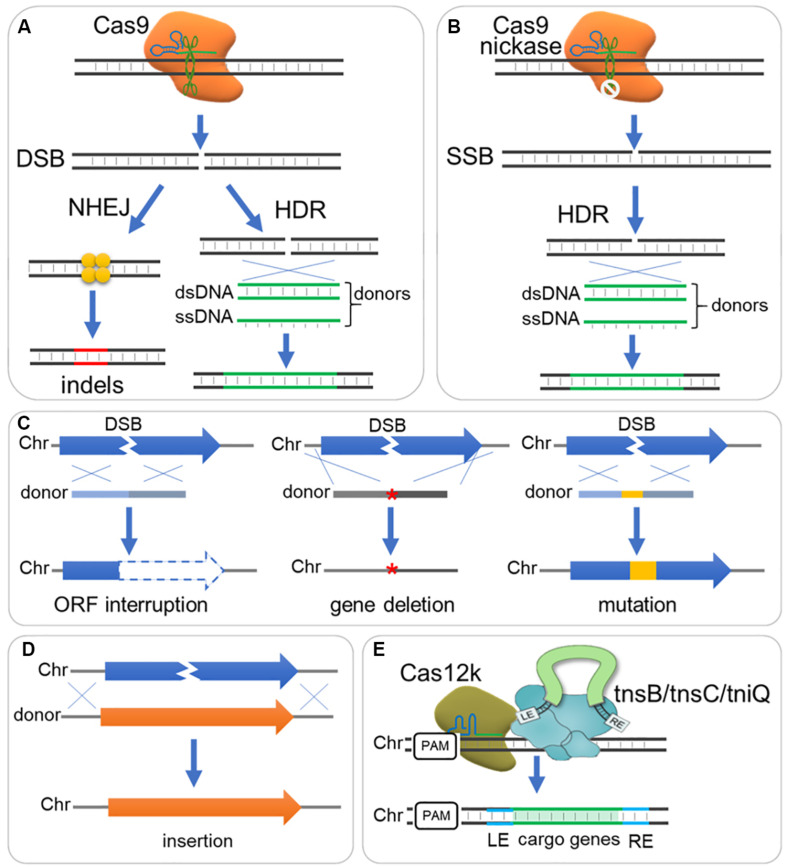
DNA repair and donor design for DNA deletion, insert and mutation. **(A)** The Cas9-sgRNA complex binds to DNA target and triggers a double strand break (DSB), which is subsequently repaired generally through non-homologous end joining (NHEJ) or homology-directed (HDR) pathway. NHEJ directs ligation of two break ends with little or no sequence homology required, resulting in small insertions or deletions (indels); while HDR repairs DSB according to a DNA template with homology sequence, resulting in precise editing when supplemented with ds- or ss-DNA donors. **(B)** A Cas9 nickase mutant with HNH or RuvC inactive domain introduces a single strand break (SSB), which can be repaired by HDR rather than NHEJ pathway. **(C)** Donor designs for gene interruption, deletion and mutation. Gene interruption: Small deletion (e.g., 8 bp) or insertion is integrated to shift reading frame, or stop codon is introduced to interrupt gene translation. Gene deletion: A donor fused with sequence upstream and downstream ORF is sued for gene deletion (‘*’ indicate the deleted gene). Gene mutation: Sequence mutations can be introduced by a donor, where seed sequence and PAM should be destroyed to avoid cutting again by Cas9-sgRNA complex. Chr, chromosome. **(D)** Donor design for sequence insertion. A donor contain long sequence is integrated through HDR, and longer homology arms are required when inserting long sequence. **(E)** Another strategy employing CRISPR I-F or V-K (e.g., Cas12k) mediates DNA integration with Tn7-like transposons (e.g., *tnsB*/*tnsC*/*tniQ*).

The CRISPR/Cas mediated precise genome editing relies on intrinsic DNA repair mechanisms after a DSB or SSB was introduced to genome by a Cas protein, e.g., Cas9 nuclease or a Cas9 mutant (Cas9 nickase, nCas9) (Figures 2A,B). There are two main pathways for DSB repair in nearly all organisms: non-homologous end-joining (NHEJ), direct ligation of two break ends with little or no sequence homology required; and homology-directed repair (HDR), repairing DSB according to a DNA template with homology sequence (Figure 2A; Ceccaldi et al., 2016; Ranjha et al., 2018). Despite alternative end joining [alt-EJ, also termed microhomology-mediated end-joining (MMEJ)] and single-strand annealing (SSA) may also repair DSBs in some organisms, NHEJ and HDR remain dominant pathways in most organisms (Ceccaldi et al., 2016; Ranjha et al., 2018). NHEJ is a fast, template independent and mutagenic pathway for DSB repair that occurs in whole cell cycle (Chang et al., 2017); whereas HDR is a slow, accurate, template dependent pathway for both DSB and SSB repair, but only occurs in S/G2 phase (Ranjha et al., 2018). NHEJ introduces unpredictable patterns of insertions and deletions, but if multiple DSBs are present, large deletions or chromosomal rearrangements may occur (Chang et al., 2017; Ranjha et al., 2018). On the other hand, CRISPR/Cas mediated precise genome editing relies on DSB or SSB repairing through HDR pathway and DNA template (donor DNA).

Both single strand DNA (ssDNA) and double strand DNA (dsDNA) fragments can be used as donors for genome editing. Despite ssDNA donors showed higher editing efficiency than dsDNA donors in several researches (Ran et al., 2013b; Miura et al., 2015; Singh et al., 2015), dsDNA donors (linear or circular) showed comparable efficiency but higher flexibility and have been widely adopted for gene deleting, mutation and insertion (DiCarlo et al., 2013; Zerbini et al., 2017; Zhang et al., 2019).

A DNA donor could be provided as HDR template to destroy the target open reading frame, and change or eliminate gRNA binding sequence and PAM to avoid repeated cleavage by Cas protein (Figures 2A,C; Raschmanova et al., 2018; Zhang et al., 2019). A short dsDNA donor, with ∼50 bp homologous sequence at each end, is usually viable and can be prepared by PCR of two oligonucleotide primers (Jakociunas et al., 2015; Zhang et al., 2019). Such short donors can also be used for introduction of single-nucleotide mutations within different gene loci (Wang Y. et al., 2016), if the locus to be edited is within the “GG” loci of a PAM or the 20 nt protospacer. Long dsDNA donors can be used for insertion (Figure 2D). Expression cassettes or other inserts can be carried by long donors and inserted to genome through HDR pathway (Figure 2D). These donors should have long homology arms (0.1–3 kb) for efficient HDR (Doetschman and Georgieva, 2017), and up to 24 kb fragments have been integrated to yeast genome through CRISPR/Cas9 system (Shi et al., 2016, 2019). Recently, transposons were proposed as an alternative tool to mediate DNA integration via a HDR independent way (Klompe et al., 2019; Strecker et al., 2019), which depends on type I–F or V-K CRISPR effectors (e.g., Cas12k) and interacts with Tn7-like transposons (e.g., *tnsB*/*tnsC*/*tniQ*) (Figure 2E).

### Adaption of CRISPR/Cas System to Non-model Microorganisms

As a powerful toolbox for genome editing and regulation, CRISPR systems are highly valued not only for model microorganisms (e.g., *E. coli*, *S. cerevisiae*), but also provide more applicable perspectives for non-model microorganisms that are difficult to be processed through traditional methods. Despite CRISPR/Cas systems have already been applied in plenty of microbial hosts (Freed et al., 2018; Raschmanova et al., 2018; Palazzotto et al., 2019; Wang and Coleman, 2019; Ng et al., 2020), it is still challenging to construct CRISPR system with high editing efficiency, and/or apply various CRISPR strategies in non-model microorganisms. In particular, lessons have also been learned that several limitations should be overcome to enable the multiplexed / genome-scale processing of CRISPR in non-model microorganisms, such as the delivery of gRNAs or Cas proteins, the genotoxic stress, etc.

One dominant challenge is active, reliable and sufficient expression of Cas protein and gRNAs in a non-model host. Due to the limited knowledge of non-conventional organisms, it is necessary to identify expression architectures ahead of CRISPR system construction. Constitutive or inducible RNAPII promoters are used for expression of Cas proteins, but RNAP III promoters should be used for sgRNA expression. In some organisms without identified RNAPIII promoters, RNAPII promoters can also be used to express gRNAs when proper strategies adopted when fusing sgRNA with special elements at each end, e.g., Hammerhead ribozyme, HDV ribozyme, and Csy4 cutting site (Jacobs et al., 2014; Nissim et al., 2014; Nowak et al., 2016; Weninger et al., 2016). Some architectures for stable episomal expression could also largely improve CRISPR efficiency, such as centromeric sequence (Cao et al., 2017, 2020) and autonomously replicating sequences (ARSs) (Gu et al., 2019).

Usually, the Cas9 form *S. pyogenes* (SpCas9) is efficient enough for genome editing in different organisms. Codon optimization is occasionally needed when the wildtype SpCas9 was not actively expressed. In some organisms, however, SpCas9 showed low efficiency or toxic effect, and repressed cell growth significantly (Ungerer and Pakrasi, 2016; Wendt et al., 2016; Jiang et al., 2017). To solve this issue, different CRISPR systems or effector variants (e.g., Cas12a) showed high editing efficiency but lower toxicity, and were applied in those organisms (Ungerer and Pakrasi, 2016; Jiang et al., 2017; Yeo et al., 2019).

On the other hand, the CRISPR aided precise, time-saving and markerless genome editing relays on introducing DSBs at DNA targets and repairing process thereafter. Thus the intrinsic DNA repairing system largely determinates editing efficiency in non-model microorganisms. DSB repairing through NHEJ pathway results in small random deletions or inserts at the site of DSB, rather than precise repairing according to a template through HDR pathway. Thus, in those NHEJ dominant species, CRISPR/Cas system can be used for just gene inactivation, but very low efficiency in precise DNA insertion, unless NHEJ is blocked, e.g., by knocking out KU70 and/or KU80 as mentioned before (Gao S. et al., 2016; Schwartz et al., 2016; Cao et al., 2018; Bae et al., 2020). In some species lacking HDR pathway, phage-derived recombinases (RecET and λ-Red) should be expressed to assist genome editing (Jiang et al., 2015; Wang B. et al., 2018). In addition, some chemical reagents can be supplemented to increase HDR efficiency, such as SCR7 (Maruyama et al., 2015), RS-1(Song et al., 2016), KU0060648, and NU7441 (Robert et al., 2015). The HDR pathway is the dominant mechanism for DSB repair in most bacteria, and NHEJ is present in some bacteria including *Mycobacterium*, *Pseudomonas*, and *Bacillus* (Weller et al., 2002; Shuman and Glickman, 2007). In most eukaryote, however, NHEJ is the dominant mechanism for DNA repairing. It is recently reported that expression of T4 DNA ligase provides efficient *in vivo* NHEJ repairing pathway in bacteria (Su et al., 2019). Donors also vary between organisms. In some cases, short (∼50 bp) homologous arms (HAs) are sufficient for HDR (Jakociunas et al., 2015; Zhang et al., 2019); while in other cases, long (∼1–3 kb) HAs are preferred (Doetschman and Georgieva, 2017).

### Efforts to Reduce Off-Target Effects

Despite Cas9 cleavages DNA target depending on a 20 nt spacer sequence of gRNA and PAM, it still potentially introduces an undesired DSB at an unintended chromosomal locus (off-target), possibly because of gRNA binding to a similar sequence elsewhere on chromosome (Fu et al., 2013; Hsu et al., 2013; O’Geen et al., 2015). The off-target effect may lead to unexpected DNA mutations, which limits the application of CRISPR in various organisms. Efforts to address this issue have been made to increase CRISPR specificity and to predict possible off-target loci on genome. A well designed gRNA would largely reduce the crisis of off-target (Wang and Coleman, 2019), and the “seed” sequence of gRNA (10–12 bp adjacent to the PAM) highly decides the Cas9 cleavage specificity (Jinek et al., 2012). To reduce the off-target risk and protect binding and cleavage activity, bioinformatic tools or websites have been developed for gRNA design, such as Cas-OFFinder^1^ (Bae et al., 2014) and CCTop^2^ (Stemmer et al., 2015). Using truncated sgRNAs (17-18 bp) showed reduced off-target effect with Cas9 nuclease and paired Cas9 nickases in human cells (Fu et al., 2014). sgRNAs with two unpaired Gs on the 5′ end also showed more sensitive to mismatches in human cells (Kim et al., 2015). Engineering of the Cas9 protein for fidelity or specificity improvement also largely reduces off-target effects: e.g., Kleinstiver et al. (2016) reported a high-fidelity variant, SpCas9-HF1 (N497A/R661A/Q695A/Q926A); Slaymaker et al. (2016) engineered several SpCas9 variants with high efficiency and specificity, e.g., eSpCas9(1.0) (K810A/K1003A/R1060A), and eSpCas9(1.1) (K848A/K1003A/R1060A); Chen J. S. et al. (2017) reported a new hyper-accurate Cas9 variant, HypaCas9 (N692A/M694A/Q695A/H698A), which demonstrated high genome-wide specificity without compromising on-target activity; Hu et al. (2018) reported an expanded PAM SpCas9 variant, xCas9 (xCas9-3.7: A262T, R324L, S409I, E480K, E543D, M694I, and E1219V), which showed much improved specificity and more broad PAM sequence, e.g., ‘NG,’ ‘GAA,’ and ‘GAT.’ A Cas9 nickase mutant (nCas9) system can also reduce off-target effect, in which a pair of guide RNAs is designed to bind to a narrow target region and thus nCas9 complexes introduce two SSBs on both strand of DNA, forming a DSB with sticky ends (Mali et al., 2013a; Ran et al., 2013a; Shen et al., 2014). Similarly, Guilinger et al. fused catalytically inactive Cas9 (dCas9) and FokI nuclease (fCas9), which produces DSB by simultaneous binding of two fCas9 monomers to the DNA target sites ∼15 or 25 base pairs apart, and resulted in at least 4-fold higher specificity than that of paired nickases (Guilinger et al., 2014; Tsai et al., 2014; Wyvekens et al., 2015).

Till now, the CRISPR/Cas system has already become the most commonly used gene editing tool for numerous species. It has become a precise, convenient and portable platform for genome editing and beyond.

## Regulation of Gene Expression by Crispr/Cas Toolbox

In addition to site-specific gene editing, the catalytically dead Cas protein (e.g., dCas9, with H840A and D10A mutation) that retained its capability to recognize and bind a target DNA sequence (Qi et al., 2013) has been developed as a multi-functional platform based on its DNA recognizing and binding properties. The CRISPR/dCas9 system has been intensively researched and applied for transcription regulation, complex metabolic engineering, directed revolution, gene target screening and activation of silent gene clusters (Lino et al., 2018; Tarasava et al., 2018; Xu and Oi, 2019). Especially, the CRISPR interference (CRISPRi) (Qi et al., 2013) and the CRISPR activation (CRISPRa) (Tanenbaum et al., 2014) that allow programmed controlling of gene expression without altering the genome, are effective tools for metabolic engineering, and are highlighted here.

### Repression of Gene Expression by dCas9 (CRISPRi)

**FIGURE 3:**
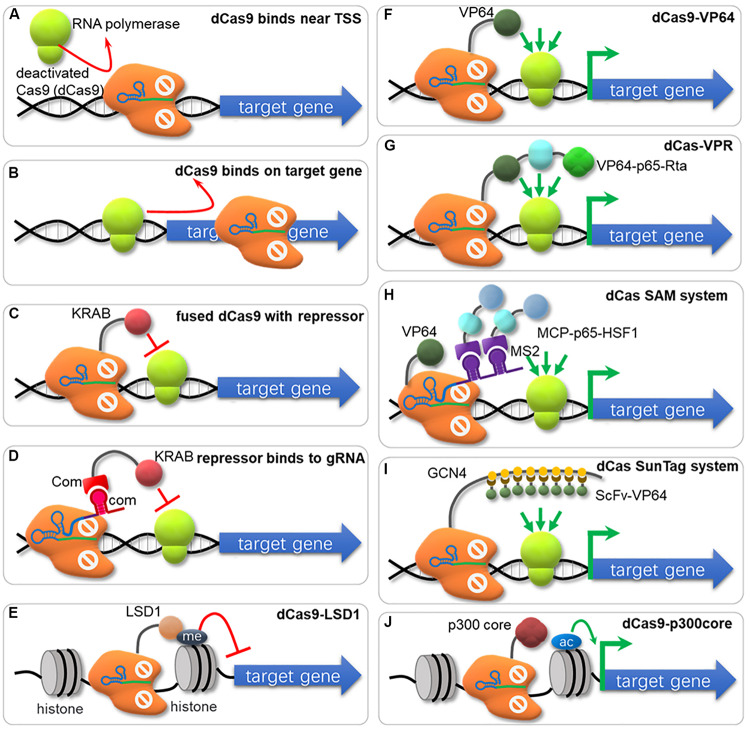
The nuclease-deactivated Cas9 (dCas9) mediated CRISPRi and CRISPRa system. **(A)** dCas9 blocks recruiting of RNA polymerase (RNAP). **(B)** dCas9 can sterically block the transcriptional elongation of RNAP. **(C)** dCas9 fuses repressors (e.g., KRAB, Mxi1) to repress gene transcription. **(D)** KRAB is fused to RNA-binding domains (e.g., COM-KRAB) and achieves gene repression when targeting DNA sites overlaps the TSS using an scaffold RNA. **(E)** Fusion of dCas9 with the histone demethylase LSD1 suppresses gene expression. **(F)** Fusion of dCas9 with activators (e.g., VP64, ω-subuint of RNAP) activates gene transcription. **(G)** The VPR strategy for gene activation. The dCas9 has been fused to the combinatory transcriptional activator VP64-p65-Rta (VPR) to amplify the activation effects. **(H)** The SAM system. The dCas9 is fused to VP64 and the sgRNA has been modified to contain two MS2 RNA aptamers to recruit the MS2 bacteriophage coat protein (MCP), which was fused to the transcriptional activators p65 and heat shock factor 1 (HSF1). **(I)** The SunTag system. The tandem repeats of a small peptide GCN4 are utilized to recruit multiple copies of scFv (single-chain variable fragment) in fusion with the transcriptional activator VP64. **(J)** dCas9 is fused with the catalytic core of the human acetyltransferase p300, which catalyzes acetylation of histone H3 lysine 27 at its target sites, corresponding with robust transcriptional activation.

CRISPR interference (CRISPRi) represses expression of targeted genes in a simple and reversible way without altered DNA sequence or off-target effects (Qi et al., 2013). Especially for those organisms lacking the RNA interference pathway, CRISPRi system offers an easy and efficient approach for targeted gene knockdown (Li et al., 2016; Peters et al., 2016). The CRISPR/dCas9 system was first used for repressing transcription by sterically hindering the RNA polymerase recruiting (Figure 3A) or RNA polymerase processivity along the coding sequence (Figure 3B; Qi et al., 2013). Ni et al. (2019) developed a CRISPRi method in which multi-gRNA plasmid was constructed that could down-regulate 7 genes simultaneously in *S. cerevisiae*. However, this ‘road blocker’ strategy using dCas9 alone is not always efficient in some organisms (Qi et al., 2013). Gilbert et al. compared different repressive effector domains, including the KRAB (Krüppel associated box) domain, the WRPW domain and the CS (Chromo Shadow) domain, and found that dCas9-KRAB was the best repressor when targeting to a window of -50 to +300 bp relative to the transcription start site (TSS), or 0–100 bp region just downstream of the TSS (Figure 3C; Gilbert et al., 2013, 2014). Another dCas9 fusion domain, Mxi1, a mammalian transcriptional repressor domain that is reported to interact with the histone deacetylase Sin3 homolog in yeast, also showed effective repression in yeast (Gilbert et al., 2013; Jensen et al., 2017; Schwartz et al., 2017; Geller et al., 2019; Wensing et al., 2019). In another research, KRAB was fused to RNA-binding domains (COM-KRAB) and achieved similar repression effects when targeting DNA sites overlapped the TSS using a scaffold RNA (scRNA) (Figure 3D; Zalatan et al., 2015). Kearns et al. fused NmdCas9 with the histone demethylase LSD1, which suppressed the expression of genes controlled by the targeted enhancers (Figure 3E; Kearns et al., 2015).

### Activation of Gene Expression by dCas9 (CRISPRa)

When dCas9 is fused with transcriptional activator and binds to the specific genomic locus, it can efficiently activate transcription via recruitment of RNA polymerase (RNAP). This CRISPR mediated transcriptional activation (CRISPRa) strategy has been applied in both prokaryotic and eukaryotic cells, and several transcriptional activators have been reported.

Bikard et al. (2013) reported a fusion protein between dCas9 and the omega subunit (ω) of RNA Polymerase (dCas9-ω) that can activate transcription by binding at an optimal distance from the promoter in *E. coli*. However, this activation effect varied depending on the binding position and the innate promoter strength, with highest activation observed for weak promoters (Bikard et al., 2013). In *S. cerevisiae*, one commonly used activator domain is VP64, consisting of four tandem copies of Herpes Simplex Viral Protein 16. dCas9-VP64 (Figure 3F) increased target gene expression by 2.5-fold, and when multiple operators were targeted, the expression reached up to 70-fold improvement (Farzadfard et al., 2013). Chavez et al. fused dCas9 with a tripartite activator VP64-p65-Rta (VPR, Figure 3G), which showed higher activating effect (∼10-fold) than dCas9-VP64 counterparts (Chavez et al., 2015). Zalatan et al. (2015) tested “scaffold RNAs” (scRNA) that encode both target locus and MS2, PP7, or com RNA hairpins, recruiting their cognate RNA-binding proteins fusing with VP64 for transcriptional activation. When the scRNA with two RNA hairpins connected by a double-stranded linker was used, stronger activation effects were observed (Zalatan et al., 2015). In a recent research, Dong et al. found that an activating effector, SoxS showed the highest effect among *E. coli* regulators (SoxS, MarA, Rob, and CAP), Hijackers (TetD, λcII, GP33, and N4_SSB_) and RNAP subunits (αNTD, RpoZ, and RpoD) in a CRISPRa system with gRNA scaffold MS2-MCP interaction in *E. coli* (Dong et al., 2018). Especially, a SoxS mutant SoxS^R93A^ and 5 aa linker further increased the activation activity (Dong et al., 2018). Konermann et al. (2015) reported a synergistic activation mediator (SAM) system for transcriptional activation (Figure 3H), which combined dCas9-VP64 with a modified scRNA system. The activator domain of p65 and the human heat shock factor 1 (HSF1) were fused with MS2 coat protein (MCP), and bound to MS2 hairpins on sgRNA for transcription activation (Konermann et al., 2015). Tanenbaum et al. developed a dCas9-SunTag system with strong activation of endogenous gene expression (Figure 3I), where the dCas9 was fused to a multimeric peptide (GCN4) array (SunTag), which can recruit multiple copies of scFv-VP64 for gene activation (Tanenbaum et al., 2014). Zhou et al. designed a new activation system, named as SunTag-p65-HSF1 (SPH), by combining the peptide array of SunTag and P65-HSF of SAM, which showed the highest level of activation compared to SAM, VPR, VP64 and SunTag in HEK293T and N2a cells (Zhou et al., 2018). Hilton et al. reported another strategy that fused dCas9 to the catalytic core of the human acetyltransferase p300 (Figure 3J). This fusion protein binds to upstream of a gene target, and catalyzes acetylation of histone H3 lysine 27 at its target sites, resulting in transcriptional activation (Hilton et al., 2015).

### Orthogonal CRISPR Systems for Comprehensive Engineering

**FIGURE 4:**
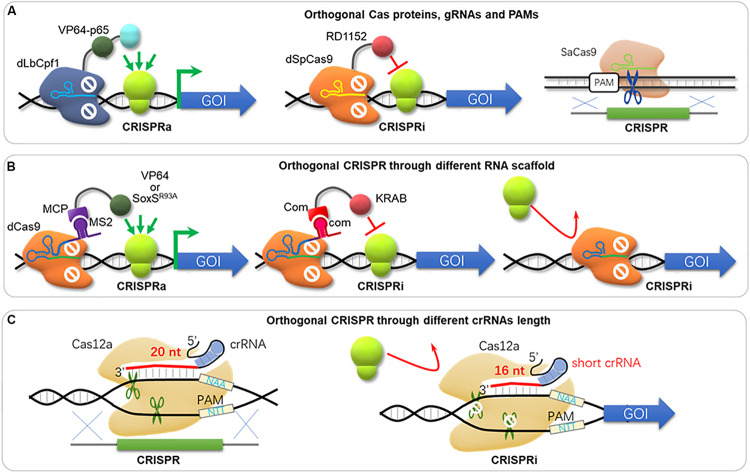
Orthogonal CRISPR systems. **(A)** The orthogonal tri-functional CRISPR system that combines transcriptional activation, transcriptional interference, and gene deletion (CRISPR-AID). This orthogonal tri-functional CRISPR system employed dLbCpf1-VP for CRISPRa, dSpCas9-RD1152 for CRISPRi, and SaCas9 for CRISPRd (gene deletion), which recognized different type of sgRNA and PAMs. dLbCpf1, dCpf1 from *Lachnospiraceae bacterium* ND2006; dSpCas9, dCas9 from *S. pyogenes*; SaCas9, Cas9 from *S. aureus*. **(B)** The orthogonal CRISPR system with different RNA scaffolds. The gRNA fused with MS2 is used for activation through binding of MCP-VP64 or MCP-SoxS^R93A^. The gRNA fused with com is used for repression through binding of Com-KRAB. Alternatively, a sgRNA without MS2 or com scaffold can hinder gene expression either. **(C)** CRISPR and CRISPRi via different crRNA length. Cas12a triggers DSB and genome editing with 20 bp-spacer in crRNA, while it blocks transcription with a short crRNA (16 bp-spacer).

In metabolic engineering and synthetic biology, complex engineering, e.g., overexpression, dynamic regulation, knock-down, and knock-out of multiple gene targets, is often required. Unfortunately, such engineering processes are often carried out sequentially and with low throughput. The development of CRISPR toolbox enables nearly all engineering types, and comprehensive applications of various CRISPR tools could solve this problem. Vanegas et al. (2017) developed a CRISPR/CRISPRi system termed SWITCH, where the Cas9 cassette was integrated into genome for genetic engineering as stage 1; and then the dCas9 cassette was integrated and replaced the Cas9 cassette for transcriptional regulation as stage 2 in *S. cerevisiae*. However, the SWITCH system does not enable genomic engineering and regulation control simultaneously. Lian et al. (2017) developed an orthogonal tri-functional CRISPR system that combines transcriptional activation, transcriptional interference, and gene deletion (CRISPR-AID, Figure 4A) in the yeast *S. cerevisiae*. This orthogonal tri-functional CRISPR system employed dLbCpf1-VP for CRISPRa, dSpCas9-RD1152 for CRISPRi, and SaCas9 for CRISPRd (gene deletion), which recognize different type of sgRNA and PAMs (Lian et al., 2017). By combining array-synthesized oligo pools, CRISPR-AID was further developed as a genome-wide system (MAGIC) to generate diversified genomic libraries to identify genetic determinants of complex phenotypes in yeast (Lian et al., 2019). This system was highlighted for complex engineering (gene interference, activation and deletion), high coverage (nearly 100% ORFs and RNA genes) and iterative/simultaneous construction, which enabled identification of new gene targets and interactions for furfural tolerance as a demonstration (Lian et al., 2019). Combining orthogonal CRISPR and CRISPRi enables genome engineering and transcriptional regulation in *E. coli*, where orthogonal Cas protein candidates were expressed for CRISPR and CRISPRi separately and simultaneously (Sung et al., 2019). Sung et al. (2019) harnessed the St1Cas9 (from *Streptococcus thermophilus*) for DNA cleavage and insertion, and the SpdCas9 for CRISPRi. In addition to orthogonal effectors, RNA scaffold and binding protein can also be used for CRISPRi and CRISPRa simultaneously. Zalatan et al. used “scaffold RNAs” (scRNA) to recruit activators or repressors (e.g., using MS2 to recruit MCP-VP64 and com to recruit Com-KRAB, Figure 4B; Zalatan et al., 2015). Thus, genes are activated or repressed depending on the scRNA features instead of Cas9 orthologs. Another strategy of simultaneous activation and interference was achieved by using one dCas9 protein but MS2 scRNAs for activation by recruiting MCP-(5aa)-SoxS^R93A^, while an unmodified gRNAs for repression (Figure 4B; Dong et al., 2018). On the other hand, one Cas12a was used for both gene editing and repression simultaneously by supplemented crRNA with different length (Figure 4C), where a 20 bp-crRNA triggers DSB and genome editing, but a 16 bp-crRNA results in gene repression without DNA cleavage (Liu W. et al., 2019).

CRISPR system can also be dynamically controlled by chemical or light with specific wavelength (ligand). Generally, a ligand induces dimerizing of two ligand binding domains (LBDs), and each domain can be fused to dCas9 and transcription effector (e.g., VPR for activation, and KRAB for repression), respectively. In such a ligand inducible CRISPRa/CRISPRi system, the presence of ligand will induce the binding of dCas9 and effector, and thus activate or repress the downstream gene expression. Several ligands have been reported for development of inducible CRISPR systems, including abscisic acid (inducing dimerization of ABI-PYL1) (Gao Y. et al., 2016; Bao et al., 2017; Chen T. et al., 2017), gibberellin (inducing dimerization of GID1-GAI24) (Gao Y. et al., 2016), rapamycin (inducing dimerization of FKBP–FRB) (Zetsche et al., 2015b; Bao et al., 2017), magnet (inducing dimerization of pMag–nMag) (Nihongaki et al., 2015a, b, 2017; Polstein and Gersbach, 2015), blue light (inducing dimerization of CRY2-CIB1), and phytochrome-based red light (inducing dimerization of PhyB–PIF (Levskaya et al., 2009). When orthogonal dCas proteins are used to response to different ligands and effector-LBDs, the CRISPR system is expected for complex, dynamic, and programmable regulations (Gao Y. et al., 2016; Bao et al., 2017; Hill et al., 2018; Xu and Oi, 2019).

### Precise Single Base Editing With CRISPR

**FIGURE 5:**
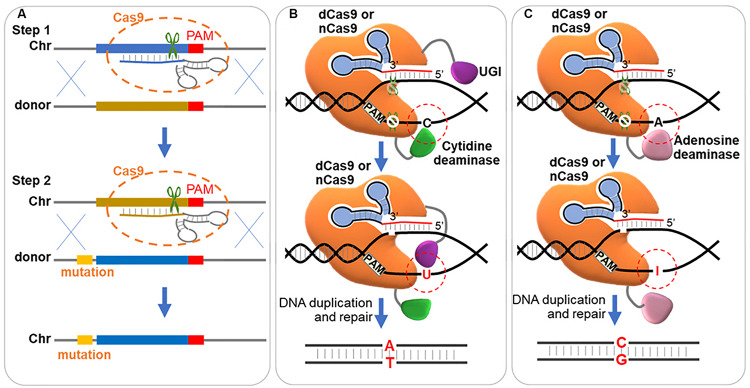
Strategies for precise single base editing. **(A)** Two-step stuffer-assisted point mutation. In the first step, a 20-nucleotide target genome sequence close to the target is replaced by a heterologous stuffer fragment via homologous recombination. In the second step, the stuffer fragment acts as the target sequence, recognized by a second gRNA, and the original sequence with arbitrary mutation is inserted back. Chr, chromosome. **(B)** ‘C→T’ mutation through DSB independent pathway. The dCas9 or Cas9 nickase (nCas9, D10A) is fused with cytidine deaminase and uracil DNA glycosylase inhibitor (UGI), and binds to a DNA target. Cytidine deaminase converts the cytidine (‘C’) to uracil (‘U’) in the non-targeted strand, which is protected by UGI from the nucleotide excision repair (NER) pathway. And in the next replication cycle, the ‘G:C’ base pair is repaired to ‘T:A’. **(C)** ‘A→G’ mutation through DSB independent pathway. The dCas9 or nCas9 is fused with adenosine deaminase and binds to a DNA target. Adenosine deaminase converts the adenosine (‘A’) to hypoxanthine (‘I’) in the non-targeted strand. And in the next replication cycle, the ‘T:A’ base pair is repaired to ‘C:G’.

Since Cas9 can tolerate mismatches in the 20 bp gRNA binding region, single-nucleotide mutations in this region could be bound and cleaved again. Thus, single-nucleotide mutations become difficult for CRISPR system. Such repeated cleavage can be avoided by introduction of additional mutations to eliminate the gRNA target site or the PAM sequence (DiCarlo et al., 2013; Jakociunas et al., 2015; Laughery et al., 2015). However, extra mutations are introduced for avoiding repeated cleavage. A two-step strategy (Figure 5A) was developed for precise single mutation by introducing the CRISPR/Cas9 twice (Biot-Pelletier and Martin, 2016; Paquet et al., 2016; Wang Y. et al., 2016). In the first step, the target was eliminated by insertion of a 20 nucleotide heterologous stuffer sequence via CRISPR/Cas9 system; and in the second step, this stuffer was eliminated by the original sequence with desired point mutation via CRISPR/Cas9 system (Biot-Pelletier and Martin, 2016; Paquet et al., 2016; Wang Y. et al., 2016).

Another method for processing precise base editing is to use dCas9 fused deaminase, which hydrolyzes the amine group of ‘C’ and ‘A,’ and enables ‘C’ to ‘T’ and ‘A’ to ‘G’ conversions without dsDNA cleavage (Figures 5B,C). Cytidine base editors (CBEs) and adenine base editors (ABEs) were developed to convert ‘C’ to ‘T’ (Komor et al., 2016) and ‘A’ to ‘G’ (Gaudelli et al., 2017) separately. Typically, BE3 (the mainly used CBE, cytidine deaminase-nCas9-UGI) and ABE7.10 (the most widely used ABE, wtTadA-mutantTadA-nCas9) showed the highest editing efficiency within the protospacer position 4–8 and 4–7 (counting the PAM as positions 21–23) (Komor et al., 2016; Gaudelli et al., 2017). CBEs using LbCpf1 showed an editing window preference of positions 10-12 (Li et al., 2018). In a recent research, a single-base editing termed CRISPR-BEST was developed by fusing Cas9 nickase (D10A) to cytidine and adenosine deaminase as editors. The CRISPR-BEST enabled ‘C→T’ and ‘A→G’ conversion within a window of approximately 7 and 6 nucleotides, respectively, with high efficiency in *Streptomyces* species (Tong et al., 2019). In another research, Zhao et al. fused dCas9 with PmCDA1 (the cytidine deaminase from *Petromyzon marinus*) and UGI (the uracil DNA glycosylase inhibitor), which enabled point mutations from ‘C’ to ‘T’ (‘C→T’) in *Streptomyces coelicolor*, and the efficiency reached up to 100%, 60%, and 20% for one, two and three loci, respectively (Zhao et al., 2019). Wang et al. fused Cas9 nickase (D10A) with activation-induced cytidine deaminase, which enabled precise ‘C→T’ conversion at one, two and three loci with an efficiency of 100%, 87%, and 23%, respectively, in *Corynebacterium glutamicum*, and built a library of 14154 unique gRNAs for inactivation of 2726 genes (Wang Y. et al., 2018). The low efficiency of triple-site editing could be possibly caused by the lower amount of the base editor at each locus than those targeting single loci. And a developed system with expanding targeting scope, editing window, and base transition capability was further constructed in *C. glutamicum* by the same group (Wang et al., 2019).

## Application of CRISPR/Cas System in Microbial Biotechnology

The fast developed and multiple functioned CRISPR system enables versatile, systematic and automatic applications in microbial technology. Especially, the CRISPR/Cas9 system has been developed for fast, efficient, precise and concise multi-loci editing and metabolic engineering. These researches imploring CRISPR/Cas system for hyper or wider applications in recent two years are shown in Table 2. And efforts for promoting CRISPR system for multi-loci editing and metabolic engineering are highlighted.

**TABLE 2:** Selected recent CRISPR mediated metabolic engineering works.

**Host**	**Toolbox**	**Product**	**Engineering by CRISPR**	**Achievements**	**References**
*Bacillus subtilis*	CRISPRi	Hyaluronic acid (HA)	Reduce the expression of *pfkA* or *zwf*	Increased HA titer of up to 108% at 2.26 g/L and enhanced molecular weight	Westbrook et al., 2018
*Bacillus subtilis*	Xylose-induced CRISPRi	N-acetylglucosamine	Reduced the expression of *zwf*, *pfkA*, *glmM*	103.1 g/L in fed-batch fermentation	Wu et al., 2018b
*Clostridium ljungdahlii*	CRISPR/Cas9	Butyric acid	A butyric acid production pathway was integrated	1.01 g/L of butyric acid within 3 days by fermenting synthesis gas (CO_2_/CO)	Huang et al., 2019
*Clostridium ljungdahlii*	CRISPRi	3-Hydroxybutyrate (3HB)	Repression of *pta* and *aor2*	Downregulation of *pta* increases 3HB production 2.3-fold with a titer at 21 mM	Woolston et al., 2018
*Clostridium tyrobutyricum*	Type I-B CRISPR-Cas	n-Butanol	Deletion of *spo0A* and *pyrF*, and integration of *adhE1* or *adhE2* to replace *cat1*	26.2g/L	Zhang et al., 2018
*Corynebacterium glutamicum*	Cas9 nickase (D10A) with activation-induced cytidine deaminase	Glutamate	Construction of a combinatorial gene inactivation library, and *pyk*/*ldhA* double inactivation for glutamate production	Increased production by 3-fold	Wang Y. et al., 2018
*Synechocystis* sp. PCC 6803	Inducible CRISPRi	n-Butanol	Repression of *gltA*	5-fold increase of carbon partitioning to n-butanol relative to a non-repression strain	Shabestary et al., 2018
*Escherichia coli*	CRISPR-Cas12a	5-Aminolevulinic acid	Integrating the *T7 RNAP* cassette and pT7-*hem1* cassette into the *lacZ* site and the *torS* site, respectively	1.55 g/L	Ao et al., 2018
*Escherichia coli*	Orthogonal CRISPR and CRISPRi systems	Succinate	Knock in *pyc*, knockout *adhE* and knockdown of *ptsG*, *ldhA*, and *pflB*	Increased by 178% with a titer at 2.5 g/L, and the titer increased to 15 g/L in a fermenter	Sung et al., 2019
*Escherichia coli*	CRISPRi	Naringenin 7-sulfate		Increased bioconversion rate by 2.83-fold (48.67%)	Chu et al., 2018
*Escherichia coli*	Iterative CRISPR EnAbled Trackable genome Engineering (iCREATE)	3HP	13 rounds of editing using iCREATE	Increased by up to 60-fold with a titer at 30 g/L	Liu et al., 2018a, b
*Escherichia coli*	PS-Brick assembly and CRISPR/Cas9	1-Propanol	*ppc*, *aspA*, *aspC*, *asd*, *pntAB*, *thrA^443^BC*, *rhtC* were overexpressed and *tdh* and *ilvA* were deleted for threonine production; *kivD* and *ADH2* were expressed in *A^443^BC* and *asd* expressed strain for 1-propanol production.	1.35 g/L in fed-batch fermentation	Liu S. et al., 2019
*Escherichia coli*	CRISPR/Cas9	Uridine	Expression of pyrimidine operon of Bacillus subtilis and *prs*, and deletion of *lacI*, *rihC*, *argF*, *thrA*, *iclR*, *purr*, *nupC* and *nupG*	70.3 g/L in fed-batch fermentation	Wu et al., 2018a
*Escherichia coli*	CRISPR/Cas9	Octanoic acid	Overexpression of *fabZ* and deletion of *fadE*, *fumAC* and *ackA*	Increased by 61% with a titer at 442 mg/L and further optimized to 1 g/L in fed-batch fermentation	Tan Z. et al., 2018
*Escherichia coli*	CRISPR/Cas9	Itaconic acid	Deletion of *ldhA*, *poxB* and *pflB*	3.06 g/L	Yang et al., 2018
*Escherichia coli*	CRISPRi	Isopentenol	Reduced expression of *asnA*, *prpE* and *gldA*	Increased by 98%	Tian et al., 2019
*Escherichia coli*	CRISPR/Cas9	Uridine		5.6 g/L	Li Y. et al., 2019
*Halomonas bluephagenesis*	CRISPR/Cas9	Poly(3-hydroxybutyrate-co-3-hydroxyvalerate) (PHBV)	Deletion of *sdhE* and *icl*	6.3 g/L cell dry weight (CDW), 65% PHBV in CDW and 25mol% 3HV in PHBV	Chen et al., 2019
*Halomonas* spp.	CRISPR/Cas9	P(3HB-co-3HV) consisting of 3-hydroxybutyrate (3HB) and 3-hydroxyvalerate (3HV)	Deletion of *prpC*	Increased 3HV fraction in the copolymers by approximately 16-folds with a fraction at 11.81 mol%	Qin et al., 2018
*Klebsiella pneumoniae*	CRISPRi	3HP	Deletion of *pmd*, *ldhA*, *aldA and mgsA*	Increased 3HP titer by 37% by reducing lactic acid synthesis, and further enhanced to 36.7 g/L 3-HP in fed-batch cultivation	Wang J. et al., 2018
*Saccharomyces cerevisiae*	GTR-CRISPR	Fatty acids	Knocking out of *FAA1*, *FAA4*, *POX1*, *ARE2*, *PAH1*, *LPP1*, *DPP1*, and *ARE1*	Increased free fatty acids by 30-fold with a titer at 559.52 mg/L, and increased total fatty acids by 1.8-fold with a titer at 943.92 mg/L	Zhang et al., 2019
*Saccharomyces cerevisiae*	CRISPRi	β-amyrin	down-regulating *ADH1*, *ADH4*, *ADH5*, *ADH6*, *CIT2*, *MLS2*, and *ERG7*	156.7 mg/L	Ni et al., 2019
*Saccharomyces cerevisiae*	CRISPRa		CRISPR/Cas-based gene activation library	Developed a CRISPR/Cas-based gene activation library, and improved thermotolerance	Li P. et al., 2019
*Saccharomyces cerevisiae*	CRISPR mediated genome shuffling			improved thermotolerance	Mitsui et al., 2019
*Saccharomyces cerevisiae*	CRISPRi and *in vivo* assembly	*cis*, *cis*-Muconic acid	Integration of multiple expression cassettes and down-regulating of *ZWF1*	Increased the titer by 5–21%	Kildegaard et al., 2019
*Saccharomyces cerevisiae*	CRISPRi, construction of tRNA-sgRNA operons using LEGO	2,3-Butanediol (BDO)	Knocking down *ADH1*/*3*/*5* and *GPD1*, and overexpression of *BDH1*	Increased BDO titer by 2-fold	Deaner et al., 2018
*Saccharomyces cerevisiae*	CRISPRa	3HP	A gRNA library targeting 168 genes	increased by 15 - 36%	Ferreira et al., 2019
*Synechocystis sp.*	CRISPRi	Fatty alcohols	Repression of aar, ado, sll1848, sll1752, slr2060, and slr1510	Increased by 3-fold with a specific titer of octadocanol at 10.3 mg/g DCW	Kaczmarzyk et al., 2018
*Ustilago maydis*	CRISPR/Cas9	Itaconic acid	Δ*cyp3*, ΔMEL, ΔUA, and Δ*P*_*ria1*_::P*_*etef*_*	Increased by 10.2-fold with a yield at 19.4 g/L and further enhanced to 53.5 g/L under optimized medium	Becker et al., 2019

### Promotion of CRISPR/Cas System for Multi-Loci Editing

One bias of CRISPR/Cas system is that the Cas/sgRNA complex can bind to more than one loci when proper sgRNAs are provided, which enables multi-loci editing simultaneously. Several groups have developed the CRISPR/Cas9 system for more efficient multi-loci editing, which makes genomic engineering more efficient, simple and convenient. *E. coli* and *S. cerevisiae* are typical model strains for prokaryotic and eukaryotic organisms, respectively, and multi-loci editing strategies are well illustrated thereby, enlightening adapted multi-loci editing strategies in other organisms (Gao S. et al., 2016; Wang J. et al., 2018; Zhang et al., 2018; Liu D. et al., 2019; Schultz et al., 2019; Tran et al., 2019; Zheng et al., 2019; Yang et al., 2020).

*E. coli* is the most intensively researched prokaryotic model microorganism, and multi-loci editing mediated by CRISPR/Cas is typical in *E. coli*. Jiang et al. expressed SpCas9 and λ-Red in *E. coli*, and achieved 3 genes disruption at an efficiency of 47% (Jiang et al., 2015). Ronda et al. expressed tracrRNA and crRNA separately, and achieved 2 genes disruption at an efficiency higher than 70% in *E. coli* (Ronda et al., 2016). Bassalo et al. (2016) developed a rapid and efficient one-step engineering method, and engineered 7 targets simultaneously with efficiencies ranging from 70 to 100%. Ao et al. (2018) expressed Cas12a instead of Cas9, resulting in the efficiency of integration of 2 loci at 40%, and the efficiency of integration of 3 loci at 20%. Sung et al. developed a method that combined orthogonal CRISPR and CRISPRi and enabled constitutive knockdown of three genes, knock-in of *pyc* and knockout of *adhE*, without compromising the CRISPRi knockdown efficiency (Sung et al., 2019).

*Saccharomyces cerevisiae* is the most intensively researched eukaryotic model microorganism, which enables highly efficient multi-loci editing because of the high HDR rate. Several multi-loci editing systems have been developed, including CRISPRm, HI-CRISPR, CasEMBLR, GTR-CRISPR (Ryan and Cate, 2014; Bao et al., 2015; Jakociunas et al., 2015, 2018a; Zhang et al., 2019). Bao et al. expressed crRNA and tracrRNA separately, and *CAN1*, *ADE2* and *LYP1* were simultaneously disrupted in 4 days with an efficiency ranging from 27 to 87%. Furthermore, another three genes were simultaneously disrupted in 6 days with 100% efficiency (Bao et al., 2015). Ryan and Cate developed a CRISPRm system, where 1–3 sgRNAs were expressed by a tRNA promoter and fused to the 3′ end of the self-cleaving HDV ribozyme for protecting the sgRNA from 5′-exonucleolytic activities, and achieved modifications of 1–3 targets with 81–100% efficiency (Ryan and Cate, 2014; Ryan et al., 2014). In another research, sgRNAs were separated by a 28 nt stem-loop sequence and cleaved by Csy4 (a bacterial endoribonuclease from *Pseudomonas aeruginosa*) to generate multiple gRNAs from a single transcript for multiple gene deletion in *S. cerevisiae* (Ferreira et al., 2018). This strategy enabled a deletion of 4 genes simultaneously with an efficiency of 96% (Ferreira et al., 2018). Jakociunas et al. (2015) developed a strategy, termed CasEMBLR, for *in vivo* assembly of gene cassettes and integrated to genome at up to 3 cleavage loci by CRISPR with high efficiency (30.6%, when optimized gRNAs were used). By using this method, 15 exogenous DNA parts were correctly assembled and integrated into 3 genomic loci for carotenoid production in one transformation (Jakociunas et al., 2015, 2018a). Kildegaard adopted similar strategy for multi-architecture assembly and insertion (Kildegaard et al., 2019). Kuivanen et al. (2018) reported a high-throughput workflow for CRISPR/Cas9 mediated combinatorial promoter replacements, and successfully edited 3 loci simultaneously with a frequency of 50%. Mans et al. used *in vitro* assembly for gRNAs expression and achieved simultaneous deletion of up to 6 genes in a single transformation step with a high efficiency at 65% (Mans et al., 2015). Zhang et al. report a gRNA-tRNA array for CRISPR-Cas9 (GTR-CRISPR) for multiplexed engineering, and simultaneously disrupted 8 genes with 87% efficiency, where gRNAs were fused with tRNA^GLY^ scaffolds and expressed in 2 quadruple arrays (Zhang et al., 2019). Besides, Zhang et al. also reported an accelerated Lightning GTR-CRISPR strategy, which saving the cloning step in *E. coli* by directly transforming the Golden Gate reaction mix (the successfully assembled plasmid contained sgRNA expression cassettes and a Cas9 expression cassette) to yeast (Zhang et al., 2019). Bao et al. developed a CRISPR-Cas9- and homology-directed-repair-assisted genome-scale engineering method named CHAnGE, to construct genetic variant libraries in yeast (Bao et al., 2018). In CHAnGE, guide sequence and the homologous recombination (HDR) template were arranged and synthesized in a single oligonucleotide, and a oligonucleotide library of 24,765 unique guide sequences targeting 6,459 ORFs was synthesized on a chip and then assembled into a vector [pCRCT, harboring iCas9, tracrRNA expression cassettes and a promoter for sgRNA expression, as reported in HI-CRISPR system (Bao et al., 2015)] to build a pool of plasmids. This plasmid pool was then used to create a genome-wide gene disruption collection, in which more than 98% of target sequences were efficiently edited with an average frequency of 82% (Bao et al., 2018). In parallel, Jakociunas et al. employed error-prone PCR to generate DNA mutant libraries as donor, and used Cas9-mediated genome integration to introduce mutations at single- or multi-loci with efficiencies reaching 98-99%, for robust directed evolution (Jakociunas et al., 2018b). Besides, large chromosomal fragment deletion methods were developed based on CRISPR/Cas9 system. Easmin developed a guide RNA-transient expression system (gRNA-TES), where two sgRNA expression fragments (locating to each end of target region on genome) and DNA donor containing CgLEU2 were co-transformed into host for a replacement of up to 500-kb regions with efficiencies of 67-100% (Easmin et al., 2019).

In multi-loci editing using CRISPR systems, co-expression of many sgRNAs often requires repetitive DNA sequences (e.g., repeated promoters/terminators and guide RNA scaffolds), which possibly triggers genetic instability and phenotype loss. Reis et al. reported a non-repetitive extra-long sgRNA arrays (ELSAs) strategy, where different promoters, terminators and the sgRNAs’ 61-nucleotide handle sequences were characterized for multiplex sgRNA expression (Reis et al., 2019). Through ELSAs, 22 sgRNAs within non-repetitive extra-long sgRNA arrays are simultaneously expressed for CRISPRi system, and repressed up to 13 genes by up to 3,500-fold in *E. coli* (Reis et al., 2019). The design of ELSAs and the identified 28 sgRNA handles that bind Cas9 can be adopted for CRISPR mediated multi-loci editing for metabolic engineering and synthetic biology applications in other organisms.

### The CRISPR/Cas Mediated Metabolic Engineering

The developing powerful CRISPR toolbox enables advanced genome editing and transcription regulation, and has become the ideal strategy for metabolic engineering, because of its advantage of ease of use, modularity, and scalability. Metabolic engineering rewrites the metabolic network through single or multiple gene manipulation, to create or improve microbial cell factories for the production of fuels, chemicals, pharmaceutics, etc. CRISPR systems have been increasingly used in metabolic engineering field for construction of microbial cell factories (Yan and Fong, 2017; Mougiakos et al., 2018; Tarasava et al., 2018), and those recent works are summarized in Table 2.

One advantage of CRISPR/Cas system is that it realizes precise genome editing at multi-loci in one transformation, without integrating a marker gene on genome for selection, and thus it would largely simplify operation steps and save time and labor in metabolic engineering works. As a proof of concept, Zhang et al. employed GTR-CRISPR to engineer lipid metabolism in *S. cerevisiae* for free fatty acid (FFA) production (Zhang et al., 2019). 8 genes in lipid metabolism were deleted through two rounds operation: *FAA1*, *FAA4*, *POX1*, and *ARE2* were deleted in the first round; and after losing the plasmid through anti-selection on 5-FOA medium, *PAH1*, *LPP1*, *DPP1*, and *ARE1* were knocked out in the second round transformation (Zhang et al., 2019). Thus, the final strain with 8-gene deletion was constructed in 10 days, which produced 559.52 mg/L FFA with 30-fold increase compared with wildtype.

Application of orthogonal CRISPR systems would also make complex metabolic engineering work simpler and more efficient, and knocking-in, knocking-out, interference and activation could be simultaneously processed for multiplex target genes. Several excellent examples for orthogonal CRISPR aided metabolic engineering were demonstrated recently (Table 2). For example, Sung et al. employed a Cas9 protein from *Streptococcus thermophilus* CRISPR1 (St1Cas9) to deliver DNA cleavage, and used the common dSpCas9 for gene interference (Sung et al., 2019). Each Cas9 recognized its cognate sgRNA, and worked orthogonally. Thus, St1Cas9 was harnessed to integrate SpdCas9 and sgRNA arrays, as well as knock in *pyc* and knockout *adhE*; whereas SpdCas9 was applied for constitutive knockdown of *ptsG*, *ldhA*, and *pflB* to eliminate competing pathways for lactate, formate, and ethanol synthesis. The final engineered strain produced 2.5 g/L succinate with 178% improvement (Sung et al., 2019).

### Other CRISPR Applications

The fast development of CRISPR tools enable various applications beyond genome editing and transcriptional regulation. One application is building activated and/or interfered gene libraries to screen phenotype related genes. Gilbert et al. applied genomic libraries of CRISPRi and CRISPRa to screen gene targets related to the sensitivity to a cholera-diphtheria toxin (Gilbert et al., 2014). Li et al. build a CRISPR/Cas-based gene activation library, and used it to screen gene targets for improved thermotolerance in *S. cerevisiae* (Li P. et al., 2019). Lee et al. used a CRISPRi system, targeting 4,565 (99.7%) genes to identify a minimal set of genes required for rapid growth of *Vibrio natriegens* (Lee et al., 2019). Bassalo et al. applied CRISPR/Cas9 to perform a parallel and high-resolution interrogation of over 16,000 mutations to identify proteins associated to lysine metabolism in *E. coli* (Bassalo et al., 2018). While Wang et al. built a larger guide RNA library of ∼60,000 members for coding and non-coding targets in *E. coli*, and applied CRISPRi system to associate genes with phenotypes at the genome level (Wang T. et al., 2018).

CRISPR system can also be used to discover novel compounds by activating the expression of silent gene or gene cluster, which may code enzymes for novel or undetectable nature products synthesis. Zhang et al. reported an one-step CRISPR/Cas9 knock-in strategy to activate biosynthetic gene cluster expression and trigger metabolite production by insertion of strong promoters upstream biosynthetic operons in *Streptomyces* species (Zhang et al., 2017; Lim et al., 2018). Grijseels et al. (2018) implemented the CRISPR/Cas9 technology to identify the decumbenone biosynthetic gene cluster in *Penicillium decumbens*, and evaluated the importance of targets for production of calbistrin. Similarly, Lee et al. (2018) adopted the CRISPRi system for rapid identification of unknown carboxyl esterase activity in *C. glutamicum*. Naseri et al. (2019) employed orthogonal, plant-derived artificial transcription factors (ATFs) for the balanced expression of multiple genes in *S. cerevisiae*, and generated CRISPR/Cas9-mediated cell libraries for producing β-carotene and co-producing β-ionone and biosensor-responsive naringenin.

CRISPR/Cas9 system also amplified the power of evolutionary engineering for industrial microorganisms. Mitsui et al. developed CRISPR/Cas9 system as a genome shuffling method for evolutionary engineering to obtain a thermotolerant mutant strain (Mitsui et al., 2019). Halperin et al. (2018) proposed a new method called EvolvR that can accelerate mutagenesis up to 7,770,000-fold within a tunable window length via CRISPR-guided nickases. Jakoèiûnas et al. reported a method named Cas9-mediated Protein Evolution Reaction (CasPER) for efficient mutagenesis of nucleotides by combining error-prone PCR and Cas9-mediated genome integration (Jakociunas et al., 2018b). Garst et al. (2017) constructed CRISPR-enabled trackable genome engineering (CREATE) method, where a library of targets was built and transformed for multiplex editing *in vivo*, followed by screening and mutation identification. Through CREATE, a library of 10^4^–10^6^ individual members was built, and an average mutation rate of 75% was reached for site saturation mutagenesis for protein engineering and adaptive laboratory evolution (Garst et al., 2017). Based on CREATE, Liu et al. developed an iterative CRISPR EnAbled Trackable genome Engineering (iCREATE) strategy for the rapid construction of combinatorially modified genomes, and used it for 3-hydroxypropionate (3HP) production improvement (Liu et al., 2018a, b).

## Conclusion and Future Perspectives

The intrinsic advantage of CRISPR enables an evolutionary and versatile platform for genotypic, metabolic and phenotypic engineering in microbial biotechnology. The CRISPR based tools are generally with higher efficiency, more convenience, more efficient multiplex targets editing/regulation and time-saving compared with traditional ones. However, challenges and weaknesses still exist. Despite the CRISPR/Cas system has been used for a broad range of microorganisms, the genome editing efficiency varies between species to species and even between cell to cell, indicating cellular intrinsic process impacts CRISPR/Cas system. More reliable, inducible and widely applicable expression architectures, e.g., RNAPII- and RNAPIII-promoters, ARSs and centromere sequences can be developed for multi-hosts, which would enable the expression of Cas effectors and gRNAs in different organisms with simple modification, especially in non-model microorganism, which would make CRISPR a portable platform and transplant CRISPR strategies from model microorganism to those non-model ones. The efficiency of CRISPR system (both for genome editing and transcriptional regulation tools) showed a gRNA position reliable phenomenon, which means high-efficiency on some gRNAs, but low-efficiency or even non-work on others. Therefore, more than one gRNAs should be tested when editing a new target, especially for efficient CRISPRa and CRISPRi. Thus, it remains important for developing more powerful effectors for robust activation/inference, engineering the Cas protein for better performance, and developing algorithms that can predict and design efficient gRNAs for CRISPRa and CRISPRi at single nucleotide level. Another highlighted direction is a comprehensive application of different CRISPR/Cas systems to facilitate insertion, deletion and transcriptional regulation simultaneously. Lian et al. developed a such strategy in *S. cerevisiae* (Lian et al., 2017), enabling perturbation of the metabolic and regulatory networks in a modular, parallel, and high-throughput manner, which is worthy to adapt such strategy in other organisms. Besides, with the genome wide application of CRISPR and array-synthesized oligo pools, it is more easier to generate large libraries containing millions and even billions of variants (Lian et al., 2019). Therefore, developing high throughput techniques, e.g., high efficient transformation methods, robotic platforms and microfluidic systems remain necessary and challenging. Furthermore, with the aid of automated robotic systems (HamediRad et al., 2019), CRISPR system could become more powerful for functional mapping and multiplex optimization of strains in an unprecedented scale.

On the other hand, types VI and III CRISPR systems were reported to have specialized or pluralistic for RNA targeting activity (Shmakov et al., 2017), which enabled direct RNA engineering by CRISPR systems (Abudayyeh et al., 2016, 2017). Despite limitations in those RNA-targeting CRISPR systems [reviewed in Smargon et al. (2020)], it has showed capabilities in RNA imaging (Abudayyeh et al., 2017), RNA interference (Abudayyeh et al., 2016), RNA mutation (Abudayyeh et al., 2017) and RNA detection (Gootenberg et al., 2017, 2018). Thus CRISPR aided RNA manipulation shows bright prospect as an emerging tool in fundamental research and bioengineering.

## Author Contributions

WD and SS outlined this manuscript. WD drafted the manuscript. SS and YZ revised the manuscript. All authors contributed to the article and approved the submitted version.

## Conflict of Interest

The authors declare that the research was conducted in the absence of any commercial or financial relationships that could be construed as a potential conflict of interest.

## Footnotes

1 http://www.rgenome.net/cas-offinder/

2 https://crispr.cos.uni-heidelberg.de
